# Supplementary material for: Evaluation in a Cytokine Storm Model In Vivo of the Safety and Efficacy of Intravenous Administration of PRS CK STORM (Standardized Conditioned Medium Obtained by Coculture of Monocytes and Mesenchymal Stromal Cells)
Source: Biomedicines. 2022 May 8;10(5):1094. doi: 10.3390/biomedicines10051094 (PMC9138962; doi:10.3390/biomedicines10051094)
Supplement: Supplementary file 1 [file biomedicines-10-01094-s001.zip › Tabla S4.pdf]

Tabla S4. Values of human cytokines analyzed by multiplex assay.

Sample	*TNF-α*	*IL-6*	*IL-10*	*IL-1β*	*IFNγ*	*IL-1RA*	*IL-12 p70*	*HGF*
16	4.353	2.492	N/A	< 15.761	16.589	N/A	50.884	6.291
17	3.410	2.153	N/A	< 15.761	17.457	105.010	63.737	7.748
18	3.099	2.237	< 4.033	< 15.761	17.457	13.938	60.530	7.754
19	3.880	2.153	N/A	< 15.761	19.194	< 26.132	57.318	6.291
20	3.571	1.901	N/A	< 15.761	17.457	2.781	50.900	7.748
21	3.566	2.153	< 4.033	< 15.761	15.720	8.360	44.465	6.258
22	3.723	2.153	N/A	< 15.761	15.719	82.099	57.318	7.748
23	3.723	1.984	< 4.033	< 15.761	15.720	2.781	63.725	6.291
24	4.040	2.153	N/A	< 15.761	13.982	5.923	54.111	7.748
25	3.410	2.321	< 4.033	< 15.761	10.502	< 26.132	50.900	4.800
26	3.413	1.986	< 4.033	< 15.761	12.243	2.781	50.884	6.291
27	3.723	2.068	< 4.033	< 15.761	13.982	50.092	63.725	7.748
28	3.880	2.321	8.244	< 15.761	16.588	8.360	57.318	6.291
29	3.566	1.984	< 4.033	< 15.761	15.720	225.939	57.318	6.291
30	3.723	2.321	< 4.033	< 15.761	20.929	N/A	50.900	6.258
Limit detection value	UNK	UNK	4.033	15.761	UNK	26.132	UNK	UNK

Values "< X" are under limit detection and are represented in the graphics as that "X" exact number. N/A means that the value is either non valid or under detection limit; therefore, it was represented as half the detection limit value for each cytokine/growth factor
