# Supplementary material for: Evaluation in a Cytokine Storm Model In Vivo of the Safety and Efficacy of Intravenous Administration of PRS CK STORM (Standardized Conditioned Medium Obtained by Coculture of Monocytes and Mesenchymal Stromal Cells)
Source: Biomedicines. 2022 May 8;10(5):1094. doi: 10.3390/biomedicines10051094 (PMC9138962; doi:10.3390/biomedicines10051094)
Supplement: Supplementary file 1 [file biomedicines-10-01094-s001.zip › Table S1.pdf]

Table S1. Molecular characterization of PRS CK STORM batch used in this experiment.

**Concentration (pg/ml)**	**PRS CK STORM**		
	High Dose	Moderate Dose	Low Dose
**BDNF**	7.81	3.9025	1.95125
**EGF**	-	-	-
**Eotaxin (CCL11)**	14.59	7.295	3.6475
**FGF-2**	26.10	13.05	6.525
**GM-CSF**	7.14	3.57	1.785
**GRO alpha (CXCL1)**	623.94	311.9675	155.98375
**HGF**	845.36	422.68	211.34
**IFN alpha**	0.05	0.025	0.0125
**IFN gamma**	-	-	-
**IL-1 alpha**	2.06	1.03	0.515
**IL-1 beta**	2.96	1.48	0.74
**IL-1RA**	57,992.28	28,996.1375	14,498.0688
**IL-2**	6.74	3.37	1.685
**IL-4**	4.54	2.2675	1.13375
**IL-5**	6.76	3.38	1.69
**IL-6**	957.12	478.5575	239.27875
**IL-7**	0.13	0.0625	0.03125
**IL-8 (CXCL8)**	4,354.24	2,177.1175	1,088.55875
**IL-9**	0.06	0.03	0.015
**IL-10**	0.24	0.1175	0.05875
**IL-12p70**	0.12	0.06	0.03
**IL-13**	-	-	-
**IL-15**	-	-	-
**IL-17A (CTLA-8)**	-	-	-
**IL-18**	7.95	3.975	1.9875
**IL-21**	-	-	-
**IL-22**	-	-	-
**IL-23**	-	-	-
**IL-27**	-	-	-
**IL-31**	-	-	-
**IP-10 (CXCL10)**	77.49	38.745	19.3725
**LIF**	96.69	48.3425	24.17125
**MCP-1 (CCL2)**	2,693.36	1,346.68	673.34
**MIP-1 alpha (CCL3)**	12.13	6.0625	3.03125
**MIP-1 beta (CCL4)**	421.19	210.595	105.2975
**NGF beta**	0.67	0.335	0.1675
**PDGF-BB**	-	-	-
**PIGF-1**	69.71	34.855	17.4275
**RANTES (CCL5)**	28.87	14.435	7.2175
**SCF**	3.46	1.7275	0.86375
**SDF-1 alpha**	1,608.00	804	402
**TNF alpha**	11.62	5.81	2.905

**TNF beta**	2.47	1.235	0.6175
**VEGF-A**	1,980.66	990.3275	495.16375
**VEGF-D**	0.14	0.07	0.035
**IGF-1**	1,675.1276	837.5638	418.7819
**TIMP-1**	59,485.2725	29,742.6362	14,871.3181
**IL-6SR**	18.1163691	9.05818457	4.52909228
**MMP-1**	20,593.5698	10,296.7849	5,148.39246

Values don’t showed are under limit detection
