# Supplementary material for: Evaluation in a Cytokine Storm Model In Vivo of the Safety and Efficacy of Intravenous Administration of PRS CK STORM (Standardized Conditioned Medium Obtained by Coculture of Monocytes and Mesenchymal Stromal Cells)
Source: Biomedicines. 2022 May 8;10(5):1094. doi: 10.3390/biomedicines10051094 (PMC9138962; doi:10.3390/biomedicines10051094)
Supplement: Supplementary file 1 [file biomedicines-10-01094-s001.zip › Table S2.pdf]

Table S2. Values of biochemical profile from mice sera.

	Alkaline phosphatase (U/l)	*Alanine aminotransferase (U/l)*	*γ-glutamyl-transferase (U/l)*	*Biliary acid (umol/l)*	*Bilirubin (umol/l)*	*Albumin (g/l)*	*Blood urea nitrogen (mmol/l)*	*Cholesterol (mmol/l)*
Standard value	39+/-25.7 CD1; 59+/-C57	99+/-86.3 CD1; 41.1+/-16.4 C57	UNK	UNK	11.973 +/-3.25	31.7 +/-4.7	13.57 +/-7.179 CD1; 11.67 +/-1.25 C57	3.89 +/-0.773
16 Day 0	36.00	78.00	5.00	0.00	6.00	38.00	9.60	4.00
17 Day 0	33.33	55.56	5.56	0.00	6.67	37.78	11.56	4.22
22 Day 0	26.92	215.38	4.81	0.96	5.77	36.54	9.81	4.23
23 Day 0	84.44	157.78	5.56	0.00	6.67	28.89	7.33	3.33
25 Day 0	64.44	35.56	5.56	0.00	6.67	11.11	6.00	3.33
26 Day 0	31.43	71.43	7.14	0.00	8.57	48.57	8.57	4.57
27 Day 0	70.00	288.00	5.00	0.00	8.00	28.00	5.60	3.60
16	26.09	34.78	10.87	0.00	13.04	21.74	5.22	5.22
17	38.00	86.00	5.00	1.00	6.00	40.00	6.60	5.20
18	35.71	70.00	3.57	62.86	4.29	40.00	5.14	5.57
19	78.00	30.00	5.00	0.00	6.00	32.00	5.40	5.00
20	7.58	27.27	7.58	0.00	6.06	30.30	4.85	5.76
21	75.00	52.08	5.21	93.75	6.25	31.25	4.79	5.63
22	84.21	57.89	6.58	1.32	13.16	13.16	6.32	4.74
23	66.67	50.00	8.33	0.00	10.00	16.67	7.67	5.00
24	77.50	130.00	6.25	2.50	7.50	32.50	5.25	5.00
25	45.00	42.50	6.25	1.25	5.00	32.50	5.50	5.75
26	42.11	26.32	4.39	0.00	5.26	38.60	6.67	5.44
27	51.43	65.71	7.14	0.00	11.43	14.29	4.86	4.86
28	57.50	390.00	6.25	45.00	7.50	30.00	5.50	4.25
29	94.29	48.57	7.14	1.43	8.57	14.29	4.86	5.14
30	42.11	68.42	6.58	0.00	7.89	31.58	6.58	5.26
