# Supplementary material for: Evaluation in a Cytokine Storm Model In Vivo of the Safety and Efficacy of Intravenous Administration of PRS CK STORM (Standardized Conditioned Medium Obtained by Coculture of Monocytes and Mesenchymal Stromal Cells)
Source: Biomedicines. 2022 May 8;10(5):1094. doi: 10.3390/biomedicines10051094 (PMC9138962; doi:10.3390/biomedicines10051094)
Supplement: Supplementary file 1 [file biomedicines-10-01094-s001.zip › Table S3.pdf]

Table S3. Values of murine cytokines analyzed by multiplex assay.

Sample	*HGF*	*TNF-α*	*IL-12 p70*	*IL-1β*	*IL-6*	*IL-10*	*IFN-γ*	*TIMP-1*
18 Day 0	3,542.422	< 2.428	< 44.856	< 230.206	< 25.226	0.877	< 13.169	1,087.913
19 Day 0	4,660.284	< 2.428	< 44.856	< 230.206	22.442	3.666	< 13.169	3,043.261
20 Day 0	3,284.495	0.791	< 44.856	< 230.206	< 25.226	3.145	< 13.169	3,732.711
21 Day 0	5,752.083	N/A	< 44.856	N/A	27.908	4.173	< 13.169	5,895.56
24 Day 0	4,370.718	< 2.428	< 44.856	N/A	103.602	4.432	< 13.169	2,778.284
28 Day 0	4,512.387	< 2.428	< 44.856	N/A	79.233	2.379	< 13.169	2,768.825
29 Day 0	4,989.191	< 2.428	< 44.856	< 230.206	19.664	3.916	< 13.169	2,847.3
30 Day 0	5,653.182	< 2.428	< 44.856	< 230.206	7.338	2.382	< 13.169	2,839.6
16	4,807.298	0.791	< 44.856	< 230.206	< 25.226	2.125	< 13.169	2,300.744
17	4,337.892	< 2.428	< 44.856	N/A	< 25.226	1.875	< 13.169	3,669.097
18	5,000.635	0.953	< 44.856	N/A	< 25.226	3.666	< 13.169	2,967.363
19	4,459.538	< 2.428	< 44.856	< 230.206	< 25.226	2.379	< 13.169	1,573.371
20	4,045.425	< 2.428	< 44.856	< 230.206	< 25.226	1.621	< 13.169	2,555.955
21	4,600.824	< 2.428	< 44.856	< 230.206	< 25.226	3.149	< 13.169	2,390.33
22	4,402.813	N/A	< 44.856	< 230.206	N/A	1.875	< 13.169	3,374.034
23	3,758.583	< 2.428	< 44.856	< 230.206	N/A	2.125	< 13.169	1,905.15
24	4,261.972	< 2.428	< 44.856	< 230.206	< 25.226	0.626	< 13.169	2,264.233
25	4,022.923	0.953	< 44.856	< 230.206	< 25.226	0.869	< 13.169	1,867.89
26	5,230.300	N/A	< 44.856	N/A	< 25.226	2.635	< 13.169	1,199.275
27	3,782.830	< 2.428	< 44.856	< 230.206	N/A	3.145	< 13.169	6,654.346
28	4,008.266	0.610	< 44.856	58.627	< 25.226	2.125	< 13.169	3,931.319
29	4,461.430	< 2.428	< 44.856	< 230.206	< 25.226	2.125	< 13.169	2,061.139
30	3,660.711	< 2.428	< 44.856	< 230.206	55.308	3.659	< 13.169	2,659.215
Limit detection value	UNK	2.428	44.856	230.206	25.226	UNK	13.169	UNK

Values "< X" are under limit detection and are represented in the graphics as that "X" exact number. N/A means that the value is either non valid or under detection limit; therefore, it was represented as half the detection limit value for each cytokine/growth factor
